# Lynch syndrome diagnostic testing pathways in endometrial cancers: a nationwide English registry-based study

**DOI:** 10.1136/jmg-2024-110231

**Published:** 2024-10-21

**Authors:** Lucy Loong, Catherine Huntley, Joanna Pethick, Fiona McRonald, Francesco Santaniello, Brian Shand, Oliver Tulloch, Shilpi Goel, Margreet Lüchtenborg, Sophie Allen, Bethany Torr, Katie Snape, Angela George, Fiona Lalloo, Gail Norbury, Diana M Eccles, Marc Tischkowitz, Antonis C Antoniou, Paul Pharoah, Adam Shaw, Eva Morris, John Burn, Kevin Monahan, Steven Hardy, Clare Turnbull

**Affiliations:** 1 Institute of Cancer Research Division of Genetics and Epidemiology, Sutton, UK; 2 National Disease Registration Service, London, UK; 3 Health Data Insight, Cambridge, UK; 4 Cancer Epidemiology and Cancer Services Research, Centre for Cancer, Society & Public Health, Comprehensive Cancer Centre, King's College London, London, UK; 5 Department of Clinical Genetics, St George's University Hospitals NHS Foundation Trust, London, UK; 6 Gynaecology Unit, Royal Marsden NHS Foundation Trust, London, UK; 7 The Institute of Cancer Research—Sutton, London, UK; 8 Clinical Genetics Service, Manchester Centre for Genomic Medicine, Central Manchester University Hospitals NHS Foundation Trust, Manchester, UK; 9 South East Genomic Laboratory Hub, Guy's and St Thomas' Hospitals NHS Trust, London, UK; 10 Human Genetics and Genomic Medicine, University of Southampton Faculty of Medicine, Southampton, UK; 11 Department of Medical Genetics, Cambridge Biomedical Research Centre, National Institute for Health Research, University of Cambridge, Cambridge, UK; 12 Centre for Cancer Genetic Epidemiology, Department of Public Health and Primary Care, University of Cambridge, Cambridge, UK; 13 Department of Computational Biomedicine, Cedars-Sinai Medical Center, Los Angeles, California, USA; 14 Department of Genetics, Guy's and St Thomas' NHS Foundation Trust, London, UK; 15 Applied Health Research Unit, Big Data Institute, Nuffield Department of Population Health, University of Oxford, Oxford, UK; 16 Translational and Clinical Research Institute, Newcastle University, Newcastle upon Tyne, UK; 17 The Lynch Syndrome and Family Cancer Clinic, St Mark's Hospital and Academic Institute, London, UK; 18 Imperial College London, London, UK

**Keywords:** databases, genetic, genetic predisposition to disease, genetic testing, gynecology, health services research

## Abstract

**ABSTRACT:**

**Background:**

For female patients with Lynch syndrome (LS), endometrial cancer (EC) is often their first cancer diagnosis. A testing pathway of somatic tumour testing triage followed by germline mismatch repair (MMR) gene testing is an effective way of identifying the estimated 3% of EC caused by LS.

**Methods:**

A retrospective national population-based observational study was conducted using comprehensive national data collections of functional, somatic and germline MMR tests available via the English National Cancer Registration Dataset. For all EC diagnosed in 2019, the proportion tested, median time to test, yield of abnormal results and factors influencing testing pathway initiation were examined.

**Results:**

There was an immunohistochemistry (IHC) or microsatellite instability (MSI) test recorded for 17.8% (1408/7928) of patients diagnosed with EC in 2019. Proportions tested varied by Cancer Alliance and age. There was an *MLH1* promoter hypermethylation test recorded for 43.1% (149/346) of patients with MLH1 protein IHC loss or MSI. Of patients with EC eligible from tumour-testing, 25% (26/104) had a germline MMR test recorded. Median time from cancer diagnosis to germline MMR test was 315 days (IQR 222–486).

**Conclusion:**

This analysis highlights the regional variation in recorded testing, patient attrition, delays and missed opportunities to diagnose LS, providing an informative baseline for measuring the impact of the national guidance from the National Institute for Health and Care Excellence on universal reflex LS testing in EC, implemented in 2020.

**WHAT IS ALREADY KNOWN ON THIS TOPIC**

- Existing evidence describes how universal reflex mismatch repair tumour testing, compared with post hoc order-initiated testing, leads to increased proportions of patients with endometrial cancer receiving testing for Lynch syndrome.

**WHAT THIS STUDY ADDS**

- This study is the first national evaluation of the diagnostic testing pathway for Lynch syndrome in patients with endometrial cancer, and the first to examine delays between diagnosis and test receipt.

**HOW THIS STUDY MIGHT AFFECT RESEARCH, PRACTICE OR POLICY**

- This study highlights regional variation, delays and missed opportunities to diagnose Lynch syndrome in England in 2019, which may also be reflective of other health systems in countries which have not moved to universal reflex testing.

## Introduction

 Lynch syndrome (LS) is characterised by an elevated genetic risk of gastrointestinal and gynaecological cancers, in particular colorectal cancer (CRC) and endometrial cancer (EC). LS is caused by germline inactivation of one of four genes acting in DNA mismatch repair (dMMR) pathways (*MLH1, MSH2, MSH6* and *PMS2),* typically due to inherited pathogenic variants (PVs).^1 2^ Early identification of patients with LS offers opportunities to reduce the chance of lethal cancers through colonoscopy surveillance, aspirin chemoprophylaxis, risk-reducing gynaecological surgeries and cascade testing of relatives.^3^,^6^ EC is one of the canonical cancers associated with LS and for many females is the first LS cancer to be diagnosed.^7^ For females with LS, the life-time risk of EC is comparable to that of CRC for *MSH2-*associated LS, and is double the risk of CRC for *MSH6*-associated LS.^1^

Tumours arising due to LS typically exhibit deficient dMMR, which can be detected via immunohistochemistry (IHC) staining and/or microsatellite instability (MSI) testing, hereafter collectively referred to as functional MMR tumour testing. IHC analysis comprises examination for loss of staining of one or more MMR proteins. MSI is a DNA manifestation of dMMR that can be quantified via a laboratory tissue assay.^8^ In series of unselected EC, approximately 26% of tumours were reported to exhibit dMMR on IHC and 22% showed MSI (either at ≥2/5 tested loci (MSI-high) or 1/5 tested loci (MSI-low)). Of these tumours showing dMMR on either IHC or MSI, approximately 10% were the result of an inherited PV in an MMR gene (ie, LS). This equates to approximately 3% of unselected EC being attributable to LS.^9^ Thus, functional MMR tumour testing can identify a subset of EC cases that are enriched for LS.

The majority of dMMR arises as a somatic (non-inherited) event. Loss of function of both alleles of an MMR gene is required to result in dMMR. In sporadic tumours with dMMR, both mutational events have arisen somatically. Somatic hypermethylation of the *MLH1* promoter region accounts for the majority of somatic events in sporadic dMMR tumours. In LS, the first mutational event is inherited and the second is somatic, typically deletion of a large region of the second allele.^10^ Performing assays for *MLH1* promoter hypermethylation in dMMR tumour samples and excluding tumours exhibiting hypermethylation can further refine the subset of EC cases that are enriched for LS.^11^

**Figure 1:**
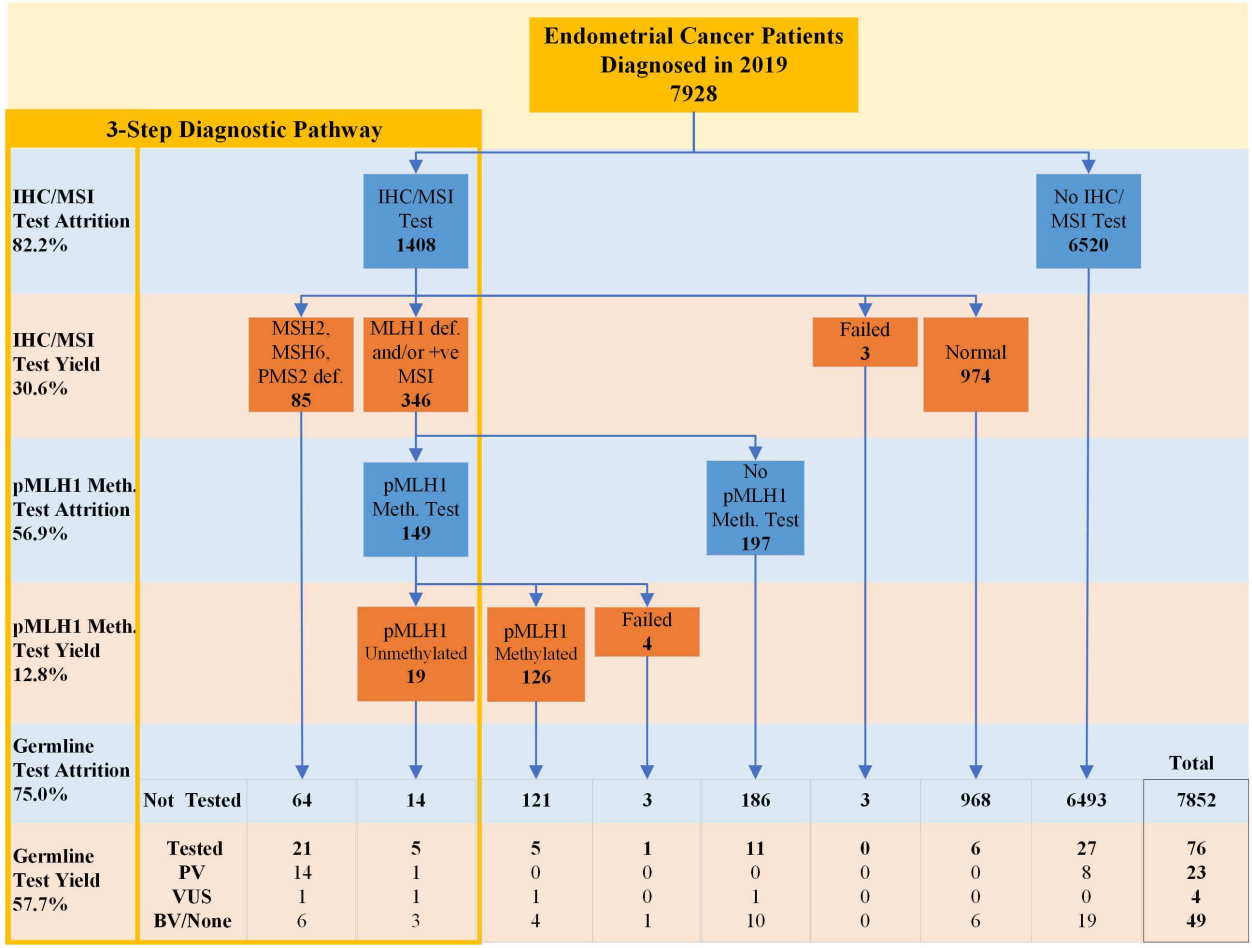
Consolidated Standards of Reporting Trials diagram showing progression of patients with endometrial cancer through the Lynch syndrome diagnostic pathway. The cohort includes patients diagnosed with endometrial cancer in 2019. The sequential three-step diagnostic pathway is depicted within the yellow outline. All patients with loss of MLH1 staining on immunohistochemistry (IHC) are included in the MLH1 def. (deficiency) category regardless of the presence or absence of other mismatch repair (MMR) protein deficits on IHC. The results of germline MMR gene testing for patients following different routes within and outside of the three-step diagnostic pathway are displayed at the bottom separated by result. Where patients have >1 test result for a test, the result is selected according to the hierarchies described in ‘Methods’ section. Attrition and yield for each of the sequential three testing steps within the diagnostic pathway are displayed on the left. Attrition: (patients with no test recorded)/(patients eligible for a test). Yield: (abnormal results)/(patients tested). Not all possible combinations of tests are shown. BV/None, benign variant or no variant identified; MSI, microsatellite instability, pMLH1 Meth. Test, *MLH1* promoter hypermethylation test; PV, pathogenic variant; VUS, variant of uncertain significance.

Thus, typically diagnostic pathways for LS testing in EC comprise three sequential steps: (i) functional MMR tumour testing (via IHC or MSI), followed by (ii) somatic *MLH1* promoter hypermethylation analysis of tumours exhibiting MLH1 protein loss on IHC or MSI and then (iii) germline genetic sequencing of MMR genes to identify PVs (figure 1). In England in October 2020, the National Institute for Health and Care Excellence (NICE) issued guidance recommending universal reflex functional MMR tumour testing in all EC.^12^ Subsequently, National Health Service England (NHSE) published a handbook to support local implementation. These documents recommend that initial tumour testing be performed using IHC as part of standard pathology, *MLH1* promoter hypermethylation should be conducted by genomic laboratories and routine germline testing should be offered by cancer treating teams, with clinical genetics managing families with PVs and conducting testing for those with more complex family histories.^13 14^ For example, patients with EC with a family history of cancer fulfilling revised Amsterdam clinical diagnostic criteria for LS may also progress to germline MMR gene testing as a first-line test.^15^ Additionally, functional MMR tumour testing may be initiated post hoc on stored histopathological tumour samples to investigate for LS in patients who were not investigated when they first presented, or for the benefit of relatives.^16^

Our recent analysis of the National Lynch Syndrome Register suggests that fewer than 5% of MMR PV carriers in England have been diagnosed.^17^ Improving the diagnosis and management of LS is an ongoing strategic priority for NHSE. This has facilitated the recent establishment of infrastructure and data pipelines for the routine submission of patient-level data for functional, somatic and germline genetic testing from National Health Service (NHS) histopathology, specialist molecular and genomic laboratories, to the National Disease Registration Service (NDRS).^13 18 19^ By linking these data at patient-level to national cancer registration records we are, for the first time, able to describe the English national landscape of known LS diagnostic testing in patients with EC in England.

The presented analyses focus on data from 2019, the inaugural year for which the national data collections made this possible and the last complete calendar year prior to the introduction of the NICE national guidelines on universal reflex LS testing in EC. The aim of the analyses was to document an informative baseline of practice in 2019 to facilitate the future evaluation of the impact of implemented national guidance. Specifically we analyse: (i) the proportions of patients undergoing testing, attrition rates and delays along the diagnostic pathway, (ii) geographic variations in delays initiating testing and proportions of patients tested and (iii) the association of sociodemographic, cancer-related and geographic factors with the likelihood of initiating testing, all within the context of the preguideline period.

## Methods

### Analyses

A retrospective national population-based observational study was conducted using data obtained from the NDRS, under an analytical partnership arrangement with the Institute of Cancer Research, London, UK. The National Cancer Registration Dataset (NCRD) covers all malignant neoplasms diagnosed from 1971 onwards.^20^ National functional MMR tumour testing (IHC and MSI) and somatic *MLH1* promoter hypermethylation testing data are available from 2019 onwards and were quality assured as complete to the best of the NDRS’ ability through to the end of 2020.^19^ National germline (constitutional) MMR gene testing data from English NHS genomic laboratories are available from 2016 onwards, with earlier data available for some regions from the year 2000.^18^ Due to database issues at genomic laboratories, a small number of germline MMR gene testing records are known to be missing from summer 2019 onwards.

The cohort for analysis comprised all patients with a malignant EC in 2019 (10th revision of the International Classification of Diseases site code C541) in the NCRD. All tumour-level functional MMR tumour testing data, tumour-level somatic *MLH1* promoter hypermethylation testing data and patient-level germline MMR gene testing data that were available within any timeframe within the NCRD as of 2 March 2024, were used to describe the number of tests recorded and results of these tests for the patient cohort.

IHC tumour analyses were considered abnormal if an abnormal or equivocal/borderline result was obtained. MSI tumour analyses were considered abnormal if an MSI-high or MSI-low result was obtained. Somatic *MLH1* promoter hypermethylation analyses were considered unmethylated (suggestive of LS), if an unmethylated or equivocal/borderline result was obtained. For abnormal germline MMR gene test records, the raw clinical genetic laboratory submission data were reviewed to verify the recorded sequence variant and the laboratory variant pathogenicity classification. Where variant pathogenicity classification was missing, the submitting laboratory was contacted for clarification.

Where multiple tests of the same type were identified for a patient, a single result was assigned to the patient according to the following hierarchy: abnormal>normal>failed/unknown for IHC and MSI tumour analyses; unmethylated (suggestive of LS)>methylated>failed/unknown for *MLH1* promoter hypermethylation or pathogenic (P)/likely pathogenic (LP)>variant of uncertain significance (VUS)>likely benign (LB)/benign (B)/no variant for germline MMR analyses. Variants classified as P and LP are hereafter referred to collectively as PV and LB and B variants collectively as benign variants.

Time (in days) was calculated from cancer diagnosis to the first of each type of test recorded for each patient. To examine geographic variation, we used the Cancer Alliance in which the patient was diagnosed. Cancer Alliances are 20 subregional, geographic, collaborative networks of NHS healthcare providers, organisations and relevant stakeholders in England.^21^ We analysed the time from cancer diagnosis to functional MMR tumour test, and difference in median time between Cancer Alliances using the Kruskal-Wallis method. Cancer Alliances are pseudonymised in the results, as we seek to investigate variation rather than calculate performance metrics in the preguideline period. Date of cancer diagnosis in NCRD is determined by the IARC Standards and Guidelines for Cancer Registration.^22^ Date of test was determined according to availability using the hierarchy: test report authorisation>test request>sample received>sample collected.

Multivariable logistic regression was performed to model the relationship between predictor variables (patient age, index of multiple deprivation (IMD) quintile, ethnicity, cancer stage, tumour grade and Cancer Alliance) and the binary outcome variable of whether there was a record of a functional MMR test having been conducted or not. Univariable logistic regression models for the six predictor variables were conducted first, with statistically significantly associated variables carried through to a multivariable model. The multivariable logistic regression model was constructed with patients with incomplete data variables excluded. For patient age category, IMD, cancer stage and grade, p values for linear trend were calculated using linear polynomial contrasts.

All analyses were completed using R Statistical Software V.4.3.1 (citations in online supplemental table 6).

## Results

7938 patients were diagnosed with malignant EC in 2019 in England. Ten patients had a recorded targeted germline MMR test for a known familial variant and/or germline MMR test before their cancer diagnosis, suggestive in both cases that these patients were previously known to clinical genetic services. These patients were excluded from the analysis. The analysed cohort therefore consisted of 7928 patients (characteristics of cohort—online supplemental table 1).

### Functional MMR tumour testing

Of the analysed cohort, 17.5% (1391 patients) were recorded as having received IHC testing and 0.7% (59 patients) were recorded as having received MSI testing. Combined, there was a record of 17.8% (1408) of patients having received at least one type of functional MMR tumour testing (0.5% (42 patients) had both IHC and MSI testing), meaning there was an 82.2% attrition of patients reaching this first step of the diagnostic testing pathway (table 1, figure 1).

**Table 1:** Total number of patients receiving functional MMR tumour testing, *MLH1* promoter methylation testing and germline MMR gene testing in the endometrial cancer cohort

Number of tests			Test results		
	Number	Per cent		Number	Per cent
**Functional MMR tumour testing**					
Immunohistochemistry	1391	17.5	Abnormal	422	30.3
			Normal	966	69.4
			Failed/Unknown	3	0.2
Microsatellite instability	59	0.7	Abnormal	25	42.4
			MSI-high	23	39
			MSI-low	2	3.4
			Normal	34	57.6
***MLH1* promoter methylation testing**					
*MLH1* promoter methylation	173	2.2	Unmethylated (suggestive of LS)	23	13.3
			Methylated	146	84.4
			Failed/Unknown	4	2.3
**Germline MMR gene testing**					
Germline MMR gene testing	76	1	Pathogenic/Likely pathogenic	23	30.3
			Variant of uncertain significance	4	5.3
			Likely benign/Benign/No variant	49	64.5

All tests and results for the cohort are counted, including those undertaken outside of the sequential three-step diagnostic testing pathway. For total tests in the cohort, percentages are presented as the number of tests/total cohort patients (7928). For test results, percentages are presented as result/number of patients tested.

LSLynch syndromeMMRmismatch repairMSImicrosatellite instability

Of patients receiving IHC testing, 30.3% (422) had an abnormal result, of which 79.9% had MLH1 deficiency (with or without PMS2, MSH2, MSH6 deficiencies), 8.8% had isolated MSH6 deficiency, 7.1% had combined MSH2 and MSH6 deficiencies, 3.3% had isolated PMS2 deficiency, 0.5% had isolated MSH2 deficiency and 0.5% had combined MSH6 and PMS2 deficiency. Of all patients receiving MSI testing, 42.4% (25) had an abnormal result (23 MSI-high and two MSI-low) (table 1).

**Figure 2:**
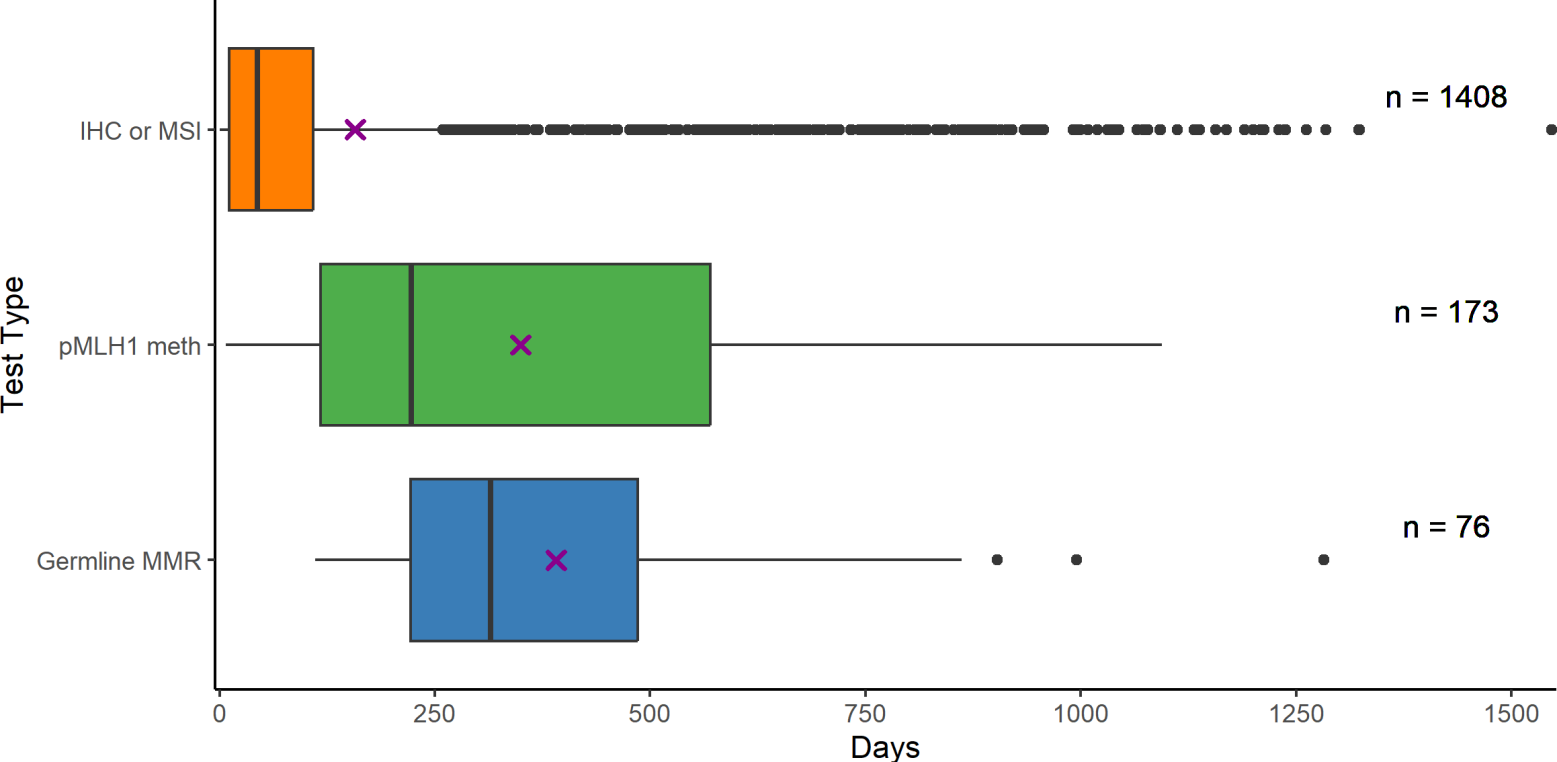
Number of days between cancer diagnosis and first test of each type. Number of days between endometrial cancer diagnosis and earliest test of each type for each patient (in days). Purple cross=mean, n=number of patients with a recorded test. IHC, immunohistochemistry; MMR, mismatch repair; MSI, microsatellite instability.

**Figure 3:**
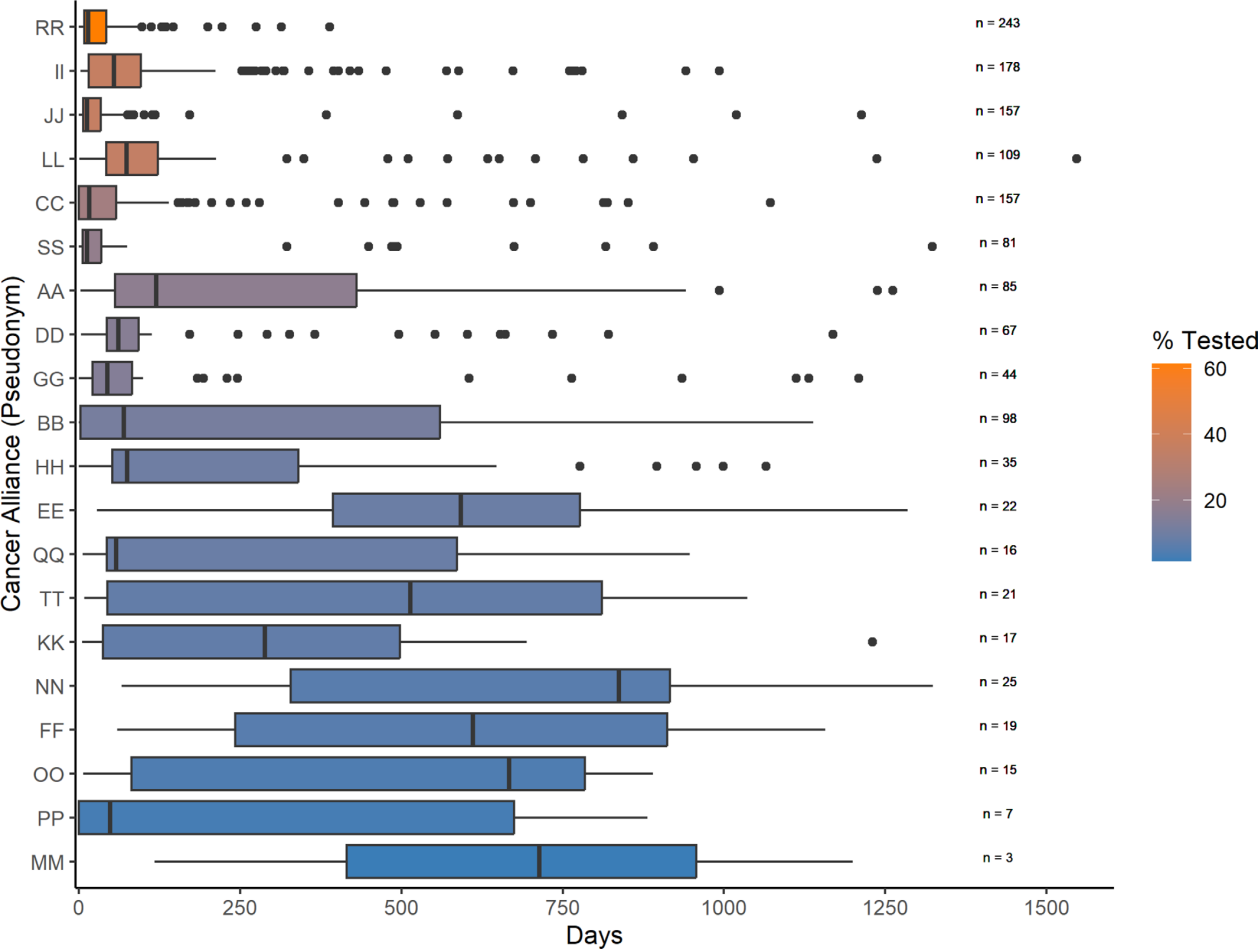
Number of days between cancer diagnosis and functional MMR tumour testing. Box plots of time in days to earliest functional MMR tumour test from date of endometrial cancer diagnosis by Cancer Alliance. n=number of patients with a recorded functional MMR tumour test within a Cancer Alliance. Fill colour=percentage of patients with a recorded functional MMR tumour test out of all patients with endometrial cancer within a Cancer Alliance (% tested) from high (orange) to low (blue). Box plots are also ordered by % tested from high (top) to low (bottom).

Median calculated time from cancer diagnosis to the earliest known functional MMR test was 44 days (IQR 11–108) (figure 2, online supplemental table 2). The median time varied significantly by Cancer Alliance, ranging from 13 days up to 837 days (Kruskal-Wallis p<0.0001). The proportion of patients diagnosed with EC that were recorded as having received functional MMR testing varied by Cancer Alliance from 1.4% to 61.2%. For 8/20 Cancer Alliances, ≥75% of the recorded functional MMR testing performed was within 4 months (122 days) from the date of cancer diagnosis (figure 3, online supplemental table 3).

To examine the factors influencing initiation of LS testing in patients with EC, univariable and multivariable logistic regression analyses were undertaken. On univariable logistic regression analyses, age at diagnosis, IMD quintile, ethnicity, stage 2 and 3 cancer, grade of tumour and Cancer Alliance were associated with the likelihood of there being a functional MMR tumour test recorded (table 2). A multivariable regression analysis was conducted using 5200 patients with complete demographic and tumour data. Following multivariable adjustment, Cancer Alliance and younger age at diagnosis (p trend <0.0001) were most strongly associated with there being a functional MMR tumour test recorded. Higher cancer grade was also associated with an increased likelihood of there being a test recorded (p trend=0.045). The associations for IMD quintile (p trend=0.083) and ethnicity were attenuated, and cancer stage largely unchanged (table 2, online supplemental table 4 and online supplemental figure 1).

**Table 2:** Univariable and multivariable logistic regression model for functional MMR tumour testing in patients with endometrial cancer

	Functional MMR test		Univariable		Multivariable n=5200		
	Not tested	Tested	OR (95% CI)	P value	OR (95% CI)	P value	P trend
**Age group (years)**							
70+	3037 (86.4)	477 (13.6)	–		–		**<0.0001**
50–69	3151 (80.9)	743 (19.1)	1.50 (1.32 to 1.70)	**<0.0001**	1.48 (1.24 to 1.77)	**<0.0001**	
30–49	323 (64.3)	179 (35.7)	3.53 (2.87 to 4.33)	**<0.0001**	6.60 (4.87 to 8.95)	**<0.0001**	
0–29	9 (50.0)	9 (50.0)	6.37 (2.47 to 16.4)	**0.0001**	14.5 (3.65 to 59.3)	**0.0001**	
**IMD quintile**							
Q1—most deprived	1209 (85.4)	207 (14.6)	–		–		0.083
Q2	1233 (79.4)	319 (20.6)	1.51 (1.25 to 1.83)	**<0.0001**	1.29 (0.97 to 1.71)	0.077	
Q3	1385 (82.7)	290 (17.3)	1.22 (1.01 to 1.49)	**0.042**	1.18 (0.89 to 1.56)	0.25	
Q4	1363 (81.3)	313 (18.7)	1.34 (1.11 to 1.63)	**0.0027**	1.52 (1.15 to 2.00)	**0.0029**	
Q5—least deprived	1330 (82.7)	279 (17.3)	1.23 (1.01 to 1.49)	**0.042**	1.21 (0.91 to 1.62)	0.18	
**Ethnicity**							
White	5444 (83.2)	1101 (16.8)	–		–		
Asian	252 (73.7)	90 (26.3)	1.77 (1.37 to 2.26)	**<0.0001**	0.55 (0.38 to 0.79)	**0.0017**	
Black	140 (70.7)	58 (29.3)	2.05 (1.49 to 2.79)	**<0.0001**	0.84 (0.48 to 1.46)	0.55	
Chinese	15 (60.0)	10 (40.0)	3.30 (1.43 to 7.28)	**0.0036**	1.72 (0.59 to 4.81)	0.31	
Mixed	28 (65.1)	15 (34.9)	2.65 (1.38 to 4.90)	**0.0025**	3.03 (1.23 to 7.45)	**0.015**	
Other	93 (75.0)	31 (25.0)	1.65 (1.08 to 2.46)	**0.017**	0.59 (0.32 to 1.05)	0.080	
**Stage at diagnosis**							
1	4189 (83.4)	836 (16.6)	–		–		0.22
2	353 (77.4)	103 (22.6)	1.46 (1.15 to 1.84)	**0.0013**	1.41 (1.03 to 1.91)	**0.027**	
3	444 (75.5)	144 (24.5)	1.63 (1.32 to 1.98)	**<0.0001**	1.75 (1.32 to 2.30)	**0.0001**	
4	408 (82.3)	88 (17.7)	1.08 (0.84 to 1.37)	0.53	1.20 (0.82 to 1.73)	0.34	
**Grade of tumour**							
G1—well differentiated	2938 (84.0)	559 (16.0)	–		–		0.045
G2—moderately differentiated	1364 (79.3)	357 (20.7)	1.38 (1.19 to 1.59)	**<0.0001**	1.51 (1.24 to 1.83)	**<0.0001**	
G3—poorly differentiated	1041 (77.8)	297 (22.2)	1.50 (1.28 to 1.75)	**<0.0001**	1.95 (1.57 to 2.43)	**<0.0001**	
G4—undifferentiated/anaplastic	33 (66.0)	17 (34.0)	2.71 (1.46 to 4.83)	**0.00098**	2.34 (0.91 to 5.71)	0.067	
**Cancer Alliance (pseudonym**)							
MM	213 (98.6)	3 (1.4)	–		–		
PP	248 (97.3)	7 (2.7)	2.00 (0.55 to 9.39)	0.32	1.73 (0.37 to 9.02)	0.48	
OO	358 (96.0)	15 (4.0)	2.97 (0.97 to 13.0)	0.088	2.42 (0.72 to 11.0)	0.19	
…	…	…	…	…	…	…	
II	317 (64.0)	178 (36.0)	39.9 (14.9 to 163)	**<0.0001**	34.9 (12.6 to 145)	**<0.0001**	
RR	154 (38.8)	243 (61.2)	112 (41.7 to 458)	**<0.0001**	210 (73.7 to 886)	**<0.0001**	

Unadjusted ORs are presented from univariable regression models including each single variable in turn. Missing data variables are excluded. Adjusted ORs are presented from a multivariable regression model including all variables in the table. Patients with missing data in any of the variables are excluded (n=2728), resulting in inclusion of 5200 patients in the multivariable model. Abbreviated results (highest and lowest ORs) are presented for the Cancer Alliance (full results available in online supplemental table 4). P values <0.05 are highlighted in bold. ORs and p values are presented to three and two significant figures, respectively.

IMDindex of multiple deprivationMMRmismatch repair

### Somatic MLH1 promoter hypermethylation testing

Of patients with MLH1 protein IHC loss or MSI in the first step of the diagnostic pathway, there was an *MLH1* promoter hypermethylation test recorded for 43.1% (149/346), meaning there was a 56.9% attrition of patients at this step. The yield for unmethylated (suggestive of LS) results was 12.8% (19/149) (figure 1).

Within the entire EC cohort, 173 patients were recorded as having received *MLH1* promoter hypermethylation testing, indicating that 24 patients had this test outside of the three-step diagnostic pathway. The median time from cancer diagnosis to *MLH1* promoter hypermethylation test was 223 days (IQR 117–570) (table 1, online supplemental table 2).

### Germline MMR gene testing

104 patients were eligible for germline MMR gene testing at the end of the three-step diagnostic pathway: 85 as a result of their MSH2, MSH6 and isolated PMS2 IHC deficiency result and 19 as a result of their unmethylated *MLH1* promoter hypermethylation result. Of these, only 25% (26/104) were recorded as having received germline MMR testing, meaning there was a 75% attrition of patients at this step. The age distribution of patients recorded as having received or not received a germline MMR gene test for which they were eligible was statistically significantly different: mean 54.5 (SD 11.1) and 62.6 (12.5), respectively, p=0.0038 (t-test). There was no statistically significant difference in the number of patients who had died within 2 years following their EC diagnosis between the two groups, p=0.55 (χ^2^ test).

The yield of PVs among patients eligible for germline MMR gene testing at the end of the three-step diagnostic pathway was 57.7% (15/26), with a notably high yield of 14/21 PVs in the MSH2/MSH6/isolated PMS2 IHC deficiency group compared with 1/5 in the unmethylated *MLH1* promoter methylation group (figure 1).

Within the entire cohort, a further 50 patients were recorded as having received germline MMR gene testing outside of the three-step diagnostic pathway. PVs were identified in 8/50 (16%) of these tests. In total, 1.0% (76/7928) of patients had a germline MMR gene test recorded following an EC diagnosis in this cohort.

Out of the 76 patients recorded as having received a germline MMR gene test in this cohort, 23 patients (30.3%) were found to have a PV, of which 12 (52.2%) were in *MSH6*, 6 (26.1%) were in *MSH2*, 3 (13.0%) were in *PMS2* and 2 (8.7%) were in *MLH1*. The median time from cancer diagnosis to germline MMR gene test was 315 days (IQR 222–486) (online supplemental tables 2,5).

## Discussion

We present an overview of the first systematically assembled national data collection of functional, somatic (tumour) and germline (constitutional) testing for MMR deficiency/LS in patients with EC. The collection of functional, somatic and germline MMR testing data as part of the NCRD provides ongoing opportunities for (i) audit of guideline adherence, (ii) review of future diagnostic pathway changes and (iii) research into endometrial and LS cancer outcomes. The data presented here describe the testing performed for all EC diagnosed in England in 2019 and reflect the testing landscape prior to the publication of NICE recommendations for universal reflex functional MMR tumour testing of all EC in 2020.^12^ The data serve as a valuable baseline for evaluating the impact of the NICE recommendations and provide an informative description of the effects of different testing practices (reflex and post hoc order-initiated) observed regionally in England in 2019.

There was significant attrition of patients progressing along the three-step diagnostic pathway. Within the diagnostic pathway, only 17.8% of patients with EC were recorded as having received functional MMR tumour testing, only 43.1% of eligible patients received *MLH1* promoter hypermethylation testing and of patients with EC eligible from tumour-testing only 25% were recorded as having received a germline MMR gene test.

Among patients receiving a germline MMR gene test at the end of the three-step diagnostic pathway, 57.7% (15/26) had a PV (consistent with a diagnosis of LS). A further 50 patients had germline MMR gene testing despite this either not being indicated by the results of the three-step diagnostic pathway or despite not having completed it. This is explained by the fact that many clinical genetics services in 2019 offered germline MMR gene testing as the first test to patients with EC with a personal or family history of cancer suggestive of LS. Among this group, a further eight patients with a PV were identified. Ten patients who were likely known to clinical genetics services prior to their EC diagnosis were excluded from the main analysis. Among them seven PV carriers were identified (online supplemental table 5). In total, 30 patients with an MMR PV were recorded among 7938 patients with EC. Thus, only approximately 12.6% (30 out of an anticipated 238) of potential MMR PV carriers were identified, given an estimated prevalence of LS in unselected EC of 3%.^9^

There was striking regional variation between Cancer Alliances in known functional MMR tumour testing, in both the proportion of EC cases tested (range 1.4%–61.2%) and the time from cancer diagnosis to testing (range 13–837 median days). In 8/20 Cancer Alliances, the majority (≥75%) of testing was performed within 4 months of diagnosis, suggestive of a policy of reflex testing at least for some patients and/or in some hospitals constituting the Cancer Alliances. These eight Cancer Alliances account for the majority of functional MMR testing (74%) conducted in this cohort. The remaining 12/20 Cancer Alliances tested a lower proportion of their EC cases with a predominantly longer time-to-test, suggestive of post hoc order-initiated testing (figure 3, online supplemental table 3). Previous studies comparing universal reflex versus post hoc order-initiated MMR tumour testing in patients with EC have shown that universal reflex testing leads to increased proportions of patients with EC receiving testing and greater uptake of downstream germline MMR gene testing.^23^,^26^

It is anticipated that implementation of the 2020 NICE recommendations will address the attrition of patients progressing along the three-step diagnostic pathway and the regional disparities in England. A baseline survey of EC (and CRC) multidisciplinary teams (MDTs) in England reported that, for example, only 62% of EC MDTs discussed MMR testing results at their meetings, only 66% stated they offered ‘universal testing’ and 88% did not have a systematic way of referring patients for germline genetic testing. The main barriers to the delivery of testing cited by those surveyed were local funding, service commissioning structures and time pressure.^13^ Another study revealed that 2 years after the release of NICE guidelines on universal diagnostic LS testing in CRC, only 44% of CRCs received functional MMR tumour testing.^19^ A concerted national collaborative effort is currently underway in England to address the variation and low testing levels for LS in patients with CRC and ECs by engaging key stakeholders, providing workforce training, addressing funding and system barriers and extending germline MMR gene test requesting to healthcare professionals outside of clinical genetics clinics.^13^ The national datasets and methods described in this analysis will be crucial for monitoring the continued implementation of the national guidelines.

Performance of functional MMR tumour testing did not appear to be associated with ethnicity or deprivation quintile following multivariable adjustment. This likely reflects the majority of testing being performed in centres undertaking reflex testing as part of the histopathological assessment, removing many of the demographic biases that influence being referred to a clinical genetics service for consideration of post hoc investigation for LS. Younger age at EC diagnosis was associated with receiving functional MMR tumour testing following multivariable adjustment, and previous studies have shown that even in healthcare systems with established universal reflex LS diagnostic testing pathways for EC, older age is a factor for non-adherence to those pathways.^27^

There were marked delays between each step of the diagnostic pathway and the total duration of the diagnostic pathway, from EC diagnosis to germline MMR test report, was a median of 315 days (IQR 222–486). Implementation of the NICE recommendations (with added clarity regarding roles and responsibilities) would be anticipated to shorten the duration of the diagnostic pathway and ultimately time to LS diagnosis for probands and their relatives. The median age of EC diagnosis in this cohort was 68 (IQR 59–75). Given the relatively high 10-year survival of EC in England of 71.6%, the identification of LS provides valuable opportunities for the prevention of further primary cancers.^7 28 29^ For example, delays in commencing aspirin prophylaxis and colonoscopy screening for female LS carriers in this age range could lead to CRCs being diagnosed at later stages, with the incidence of CRC between 60 and 70 years of age being 11.9% and 15.7% for *MLH1* and *MSH2* female PV carriers, respectively.^1^

The 2020 NICE guidance for EC recommends the use of IHC not MSI, based on the higher sensitivity of IHC and health economic modelling analysis.^12 30^ These analyses offer support to that recommendation. In addition to demonstrating dMMR, IHC provides additional information regarding the specific gene causing dMMR. In this analysis, 20.1% (85/422) of tumours with IHC abnormality had MSH2/MSH6/isolated PMS2 loss. These patients did not require the additional step (and attrition and time delay it entails) of *MLH1* promoter hypermethylation analysis, compared with if their dMMR had been diagnosed via MSI analysis. Yield for PVs among patients with MSH2/MSH6/isolated PMS2 IHC loss was higher (66.7%, 14/21) compared with those with MLH1 IHC loss or MSI with unmethylated *MLH1* promoter methylation status (20%, 1/5), consistent with the higher penetrance for EC in *MSH6-*associated and *MSH2*-associated LS.^1^

Limitations of these analyses include reliance on completeness and comparability of local data submissions. That a small number of germline MMR gene test records are known to be missing from summer 2019 onwards means that the proportion of patients who received germline tests may be slightly underestimated. However, in a comparable analysis of the CRC LS diagnostic testing pathway using Danish registry data, 20% of eligible patients received genetic counselling/germline testing, which is comparable to the 25% we demonstrate here.^31^ Inclusion of potentially nationally incomplete functional MMR tumour testing data beyond the end of 2020 allowed the demonstration of post hoc order-initiated tumour testing further away from the original EC diagnosis. However, it means that the amount of post hoc order-initiated testing could be underestimated. It is likely that post hoc testing makes up only a small amount of all functional MMR tumour testing conducted in EC, as it usually requires the referral of the patient to a clinical genetics service. 84.4% (1224/1450) of the functional MMR tumour tests recorded in this analysis were conducted within 1 year of the date of EC diagnosis (online supplemental table 7). The relatively small numbers of EC diagnosed in each hospital trust necessitated analysis by Cancer Alliance. Therefore, hospital trust-level variation in practice is not captured in these analyses. Lastly, personal or family history for LS-related cancers were not captured in these analyses.

Overall, there is a baseline picture, prior to the publication of the NICE national guidelines on universal reflex functional MMR tumour testing in EC, of considerable missed opportunity, delay and regional variation in the diagnosis of LS among patients with EC. This may also be reflective of missed diagnostic opportunities in other health systems in countries that have not moved to universal reflex testing. The results of these analyses provide an important baseline for measuring the impact of implementing the NICE national guidelines on the number of patients tested, testing delays, attrition of patients along the testing pathway and regional variation. Furthermore, the datasets and methods in this study facilitate ongoing assessment of diagnostic yield and provide opportunities to evaluate cancer outcomes by functional phenotypes and somatic and germline genotypes—key for continuously appraising the diagnostic accuracy and cost-effectiveness of implemented testing pathways.

## supplementary material

10.1136/jmg-2024-110231 online supplemental file 1

## Data Availability

Data are available on reasonable request. All data relevant to the study are included in the article or uploaded as supplementary information.
