# Supplementary material for: Lynch syndrome diagnostic testing pathways in endometrial cancers: a nationwide English registry-based study
Source: J Med Genet. 2024 Oct 21;61(12):e110231. doi: 10.1136/jmg-2024-110231 (PMC11671912; doi:10.1136/jmg-2024-110231)

Lynch Syndrome diagnostic testing pathways in endometrial cancers: a nationwide English registry-based study

Table of Contents

Supplementary Table 1	Characteristics of the endometrial cancer cohort
Supplementary Table 2	Time to test in days from date of endometrial cancer diagnosis to earliest test of each type for each patient
Supplementary Table 3	Time to test in days from date of endometrial cancer diagnosis to earliest functional MMR test
Supplementary Table 4	Full univariable and multivariable logistic regression model for functional MMR testing
Supplementary Table 5	Germline variants identified in the endometrial cancer cohort
Supplementary Table 6	Software package citations
Supplementary Table 7	Tests conducted within 1 year of date of endometrial cancer diagnosis
Supplementary Figure 1	Multivariable Logistic Regression OR plot (Patients with incomplete data variables excluded)

Supplementary Table 1. Characteristics of the endometrial cancer cohort

	Patients	Percent
**Gender**		
Female	7928	100
**Age at Diagnosis**		
0-29	18	0.2
30-49	502	6.3
50-69	3894	49.1
70+	3514	44.3
**Index of Multiple Deprivation Quintile**		
1 - most deprived	1416	17.9
2	1552	19.6
3	1675	21.1
4	1676	21.1
5 - least deprived	1609	20.3
**Ethnicity**		
Asian (Indian, Pakistani, Bangladeshi, Any other Asian background)	342	4.3
Black (Caribbean, African, Any other Black background)	198	2.5
Chinese	25	0.3
Mixed (White and Black Caribbean, White and Black African, White and Asian, Any other mixed background)	43	0.5
Other (Any other ethnic group)	124	1.6
Unknown (Not Known, Not Stated, Null)	651	8.2
White ((White) British, (White) Irish, Any other White background)	6545	82.6
**Cancer Stage**		
1	5025	63.4
2	456	5.8
3	588	7.4
4	496	6.3
Unknown	1363	17.2
**Cancer Grade**		
G1 - Well differentiated	3497	44.1
G2 - Moderately differentiated	1721	21.7
G3 - Poorly differentiated	1338	16.9
G4 - Undifferentiated/Anaplastic	50	0.6
Unknown	1322	16.7
**Cancer Alliance (Pseudonym)**		
AA	486	6.1
BB	848	10.7
CC	663	8.4
DD	430	5.4
EE	248	3.1
FF	361	4.6
GG	301	3.8
HH	361	4.6
II	495	6.2
JJ	437	5.5
KK	239	3
LL	310	3.9
MM	216	2.7
NN	454	5.7
OO	373	4.7
PP	255	3.2
QQ	188	2.4
RR	397	5
SS	428	5.4
TT	287	3.6
Unknown	151	1.9

Supplementary Table 2. Time to test in days from date of endometrial cancer diagnosis to earliest test of each type for each patient.

	Time to test (days)						
	**Minimum**	**Q1**	**Mean**	**Median**	**Q3**	**Maximum**	**Number**
**Functional MMR Test (IHC or MSI)**	0	11	157.6	44	108.25	1547	1408
**MLH1 promoter hypermethylation**	7	117	349.6	223	570	1094	173
**Germline MMR Test**	111	221.75	391.1	315	485.5	1282	76

Supplementary Table 3 - Time to test in days from date of endometrial cancer diagnosis to earliest functional MMR test (Immunohistochemistry or Microsatellite Instability), and proportion of patients with endometrial cancer who received functional MMRd testing by Cancer Alliance. Table ordered by proportion tested (highest to lowest).

	Time to test (days)						Proportion tested		
**Cancer Alliance (Pseudonym)**	**Minimum**	**Q1**	**Mean**	**Median**	**Q3**	**Maximum**	**Total Patients with Endometrial Cancer**	**Received Functional MMR testing**	**%**
RR	0	8	32.3	14	42	389	397	243	61.2
II	-1	14.25	115.2	54	95.25	993	495	178	36.0
JJ	0	7	48.8	13	34	1213	437	157	35.9
LL	1	42	152.6	74	122	1547	310	109	35.2
CC	0	0	82.4	16	58	1072	663	157	23.7
SS	0	6	88.5	13	35	1323	428	81	18.9
AA	2	56	272.7	120	430	1262	486	85	17.5
DD	3	43	148.5	61	92.5	1169	430	67	15.6
GG	0	21.5	176.8	44	82.5	1209	301	44	14.6
BB	0	2.25	260.5	70	559.5	1138	848	98	11.6
HH	0	51.5	256.7	75	340	1065	361	35	9.7
EE	28	394	573.3	592	777	1285	248	22	8.9
QQ	6	43.25	305.6	58	585.75	947	188	16	8.5
TT	8	44	442.0	514	811	1036	287	21	7.3
KK	5	37	316.9	288	498	1230	239	17	7.1
NN	66	328	696.8	837	916	1324	454	25	5.5
FF	59	242	576.5	611	912	1157	361	19	5.3
OO	7	81.5	501.4	667	784	890	373	15	4.0
PP	0	0	325.4	48	674.5	881	255	7	2.7
MM	117	415.5	677.0	714	957	1200	216	3	1.4
Unknown	0	6	147.2	55	103	611	151	9	6.0

Supplementary Table 4 - Univariable and multivariable logistic regression model for functional MMR tumour testing in patients with endometrial cancer. Unadjusted ORs are presented from univariable regression models including each single variable in turn. Missing data variables are excluded. Adjusted ORs are presented from a multivariable regression model including all variables in the table. Patients with missing data in any of the variables are excluded (n=2728) resulting in inclusion of 5200 patients in the multivariable model.

	Functional MMR Test		Univariable	Multivariable n = 5200, C-statistic = 0.806	
	Not tested	Tested	OR (95% CI, p-value)	OR (95% CI, p-value)	p-trend
**Age group**					
Age 70+	3037 (86.4)	477 (13.6)	-	-	p<0.0001
Age 50-69	3151 (80.9)	743 (19.1)	1.50 (1.32-1.70, p<0.0001)	1.48 (1.24-1.77, p<0.0001)	
Age 30-49	323 (64.3)	179 (35.7)	3.53 (2.87-4.33, p<0.0001)	6.60 (4.87-8.95, p<0.0001)	
Age 0-29	9 (50.0)	9 (50.0)	6.37 (2.47-16.39, p=0.0001)	14.52 (3.65-59.32, p=0.0001)	
**IMD Quintile**					
IMD Q1 - Most deprived	1209 (85.4)	207 (14.6)	-	-	p=0.0834
IMD Q2	1233 (79.4)	319 (20.6)	1.51 (1.25-1.83, p<0.0001)	1.29 (0.97-1.71, p=0.0770)	
IMD Q3	1385 (82.7)	290 (17.3)	1.22 (1.01-1.49, p=0.0424)	1.18 (0.89-1.56, p=0.2496)	
IMD Q4	1363 (81.3)	313 (18.7)	1.34 (1.11-1.63, p=0.0027)	1.52 (1.15-2.00, p=0.0029)	
IMD Q5 - Least deprived	1330 (82.7)	279 (17.3)	1.23 (1.01-1.49, p=0.0422)	1.21 (0.91-1.62, p=0.1805)	
**Ethnicity**					
White	5444 (83.2)	1101 (16.8)	-	-	
Asian	252 (73.7)	90 (26.3)	1.77 (1.37-2.26, p<0.0001)	0.55 (0.38-0.79, p=0.0017)	
Black	140 (70.7)	58 (29.3)	2.05 (1.49-2.79, p<0.0001)	0.84 (0.48-1.46, p=0.5473)	
Chinese	15 (60.0)	10 (40.0)	3.30 (1.43-7.28, p=0.0036)	1.72 (0.59-4.81, p=0.3097)	
Mixed	28 (65.1)	15 (34.9)	2.65 (1.38-4.90, p=0.0025)	3.03 (1.23-7.45, p=0.0148)	
Other	93 (75.0)	31 (25.0)	1.65 (1.08-2.46, p=0.0173)	0.59 (0.32-1.05, p=0.0805)	
**Stage at diagnosis**					
Stage 1	4189 (83.4)	836 (16.6)	-	-	p=0.2168
Stage 2	353 (77.4)	103 (22.6)	1.46 (1.15-1.84, p=0.0013)	1.41 (1.03-1.91, p=0.0272)	
Stage 3	444 (75.5)	144 (24.5)	1.63 (1.32-1.98, p<0.0001)	1.75 (1.32-2.30, p=0.0001)	
Stage 4	408 (82.3)	88 (17.7)	1.08 (0.84-1.37, p=0.5294)	1.20 (0.82-1.73, p=0.3426)	
**Grade of tumour**					
G1 - Well differentiated	2938 (84.0)	559 (16.0)	-	-	p=0.0446
G2 - Moderately differentiated	1364 (79.3)	357 (20.7)	1.38 (1.19-1.59, p<0.0001)	1.51 (1.24-1.83, p<0.0001)	
G3 - Poorly differentiated	1041 (77.8)	297 (22.2)	1.50 (1.28-1.75, p<0.0001)	1.95 (1.57-2.43, p<0.0001)	
G4 - Undifferentiated/Anaplastic	33 (66.0)	17 (34.0)	2.71 (1.46-4.83, p=0.0010)	2.34 (0.91-5.71, p=0.0666)	
**Cancer alliance pseudonym**					
MM	213 (98.6)	3 (1.4)	-	-	
PP	248 (97.3)	7 (2.7)	2.00 (0.55-9.39, p=0.3179)	1.73 (0.37-9.02, p=0.4812)	
OO	358 (96.0)	15 (4.0)	2.97 (0.97-12.95, p=0.0875)	2.42 (0.72-11.02, p=0.1892)	
FF	342 (94.7)	19 (5.3)	3.94 (1.32-16.93, p=0.0286)	2.44 (0.74-11.01, p=0.1816)	
NN	429 (94.5)	25 (5.5)	4.14 (1.43-17.52, p=0.0212)	4.64 (1.52-20.24, p=0.0162)	
KK	222 (92.9)	17 (7.1)	5.44 (1.79-23.53, p=0.0075)	3.71 (1.06-17.20, p=0.0563)	
TT	266 (92.7)	21 (7.3)	5.61 (1.90-23.96, p=0.0057)	4.46 (1.44-19.62, p=0.0202)	
QQ	172 (91.5)	16 (8.5)	6.60 (2.16-28.73, p=0.0030)	6.45 (1.95-29.28, p=0.0053)	

EE	226 (91.1)	22 (8.9)	6.91 (2.35-29.50, p=0.0019)	5.64 (1.86-24.54, p=0.0065)	
HH	326 (90.3)	35 (9.7)	7.62 (2.70-31.91, p=0.0008)	8.13 (2.79-34.69, p=0.0007)	
BB	750 (88.4)	98 (11.6)	9.28 (3.44-37.99, p=0.0002)	7.45 (2.68-31.03, p=0.0009)	
GG	257 (85.4)	44 (14.6)	12.16 (4.36-50.61, p<0.0001)	10.74 (3.67-45.93, p=0.0001)	
DD	363 (84.4)	67 (15.6)	13.10 (4.80-54.03, p<0.0001)	9.16 (3.21-38.67, p=0.0003)	
AA	401 (82.5)	85 (17.5)	15.05 (5.55-61.82, p<0.0001)	13.18 (4.71-55.10, p<0.0001)	
SS	347 (81.1)	81 (18.9)	16.57 (6.10-68.15, p<0.0001)	12.33 (4.35-51.82, p<0.0001)	
CC	506 (76.3)	157 (23.7)	22.03 (8.24-89.89, p<0.0001)	18.90 (6.87-78.33, p<0.0001)	
LL	201 (64.8)	109 (35.2)	38.50 (14.21-158.13, p<0.0001)	42.65 (15.28-178.12, p<0.0001)	
JJ	280 (64.1)	157 (35.9)	39.81 (14.84-162.74, p<0.0001)	43.39 (15.53-181.27, p<0.0001)	
II	317 (64.0)	178 (36.0)	39.87 (14.90-162.75, p<0.0001)	34.89 (12.59-145.18, p<0.0001)	
RR	154 (38.8)	243 (61.2)	112.03 (41.71-458.33, p<0.0001)	209.81 (73.72-886.11, p<0.0001)	

Supplementary Table 5 - Germline variants identified in the endometrial cancer cohort. Patients were exlcuded from the analysis if they received a targeted germline MMR test for a familial variant, and/or received germline MMR testing prior to their endometrial cancer diagnosis.

Gene	Clinical transcript (RefSeq)	Variant	Laboratory Record Classification	Laboratory Communication	Final Variant Classification		
MLH1	NM_000249.3	c.156del	5		5		
MLH1	NM_000249.3	c.677G>T	4		4		
MSH2	NM_000251.2	c.(942+1_943-1)(1386+1_1387-1)del	5		5		
MSH2	NM_000251.2	exon1del	5		5		
MSH2	NM_000251.2	c.942+1G>A	5		5		
MSH2	NM_000251.2	c.942+3A>T	5		5		
MSH2	NM_000251.2	c.942+3A>T	5		5		
MSH2	NM_000251.2	c.1705_1706del	5		5		
MSH6	NM_000179.2	c.3220_3221del	5		5		
MSH6	NM_000179.2	c.24C>T	5		5		
MSH6	NM_000179.2	c.3932_3935dup	5		5		
MSH6	NM_000179.2	c.3261dup	5		5		
MSH6	NM_000179.2	c.3939_3957del	NA	Pathogenic	5		
MSH6	NM_000179.2	c.3640G>T	5		5		
MSH6	NM_000179.2	c.394_395del	5		5		
MSH6	NM_000179.2	c.718C>T	5		5		
MSH6	NM_000179.2	exons3-9del	NA	Pathogenic	5		
MSH6	NM_000179.2	c.1444C>T	5		5		
MSH6	NM_000179.2	c.3622dup	5		5		
MSH6	NM_000179.2	c.3514_3515insT	5		5		
PMS2	NM_000535.5	exons9-10del	5		5		
PMS2	NM_000535.5	c.137G>T	5		5		
PMS2	NM_000535.5	exons9-10del	5		5		
MLH1	NM_000249.3	c.1976G>A	3		3		
MSH6	NM_000179.2	c.3727A>T	3		3		
MSH6	NM_000179.2	c.2341C>T	3		3		
PMS2	NM_000535.5	c.961G>A	3		3		
**Variants in patients excluded from analysis**						**Reason for Exclusion**	
MSH2	NM_000251.2	c.1213_1217dupTACCG	NA	Pathogenic	5	Targeted	Prediagnosis
MSH2	NM_000251.2	c.1189C>T	5		5	Targeted	Prediagnosis
MLH1	NM_000249.3	c.1149G>A	3		3	Fullscreen	Prediagnosis
MSH2	NM_000251.2	c.998G>A	5		5	Fullscreen	Prediagnosis
MSH2	NM_000251.2	c.1861C>T	5		5	Targeted	Prediagnosis
MSH6	NM_000179.2	c.3582_3585dupAAGT	5		5	Targeted	Prediagnosis
MSH2	NM_000251.2	exons11-16del	NA	Pathogenic	5	Targeted	Prediagnosis
PMS2	NM_000535.5	exons9-10del	5		5	Targeted	Postdiagnosis

Supplementary Table 6 - Software package citations

Package Name	Citation
R version 4.3.1 (2023-06-16 ucrt)	R Core Team (2021). R: A language and environment for statistical computing. R Foundation for Statistical Computing, Vienna, Austria. URL https://www.R-project.org/.
R Studio	Posit team (2023). RStudio: Integrated Development Environment for R. Posit Software, PBC, Boston, MA. URL http://www.posit.co/.
tidyverse	Wickham H, Averick M, Bryan J, Chang W, McGowan LD, François R, Grolemund G, Hayes A, Henry L, Hester J, Kuhn M, Pedersen TL, Miller E, Bache SM, Müller K, Ooms J, Robinson D, Seidel DP, Spinu V, Takahashi K, Vaughan D, Wilke C, Woo K, Yutani H (2019). "Welcome to the tidyverse." _Journal of Open Source Software_, *4*(43), 1686. doi:10.21105/joss.01686 <https://doi.org/10.21105/joss.01686>.
finalfit	Harrison E, Drake T, Pius R (2024). _finalfit: Quickly Create Elegant Regression Results Tables and Plots when Modelling_. R package version 1.0.71, <https://github.com/ewenharrison/finalfit>.
GGally	Schloerke B, Cook D, Larmarange J, Briatte F, Marbach M, Thoen E, Elberg A, Crowley J (2024). _GGally: Extension to 'ggplot2'_. R package version 2.2.1, <https://CRAN.R-project.org/package=GGally>.
broom	Robinson D, Hayes A, Couch S (2023). _broom: Convert Statistical Objects into Tidy Tibbles_. R package version 1.0.5, <https://CRAN.R-project.org/package=broom>.

Supplementary Table 7 - Tests conducted within 1 year of date of endometrial cancer diagnosis

TEST	Tests Recorded Within 365 Days of Endometrial Cancer Diagnosis		Total Tests Recorded for Cohort
	Number	%	
Immunohistochemistry	1192	85.7	1391
Microsatellite Instability	32	54.2	59
MLH1 Promoter Methylation	107	61.8	173
Germline MMR	27	35.5	76

Supplementary Figure 1 - Multivariable Logistic Regression OR plot for functional MMR testing in endometrial cancer patients diagnosed in 2019. (Patients with incomplete data variables excluded)

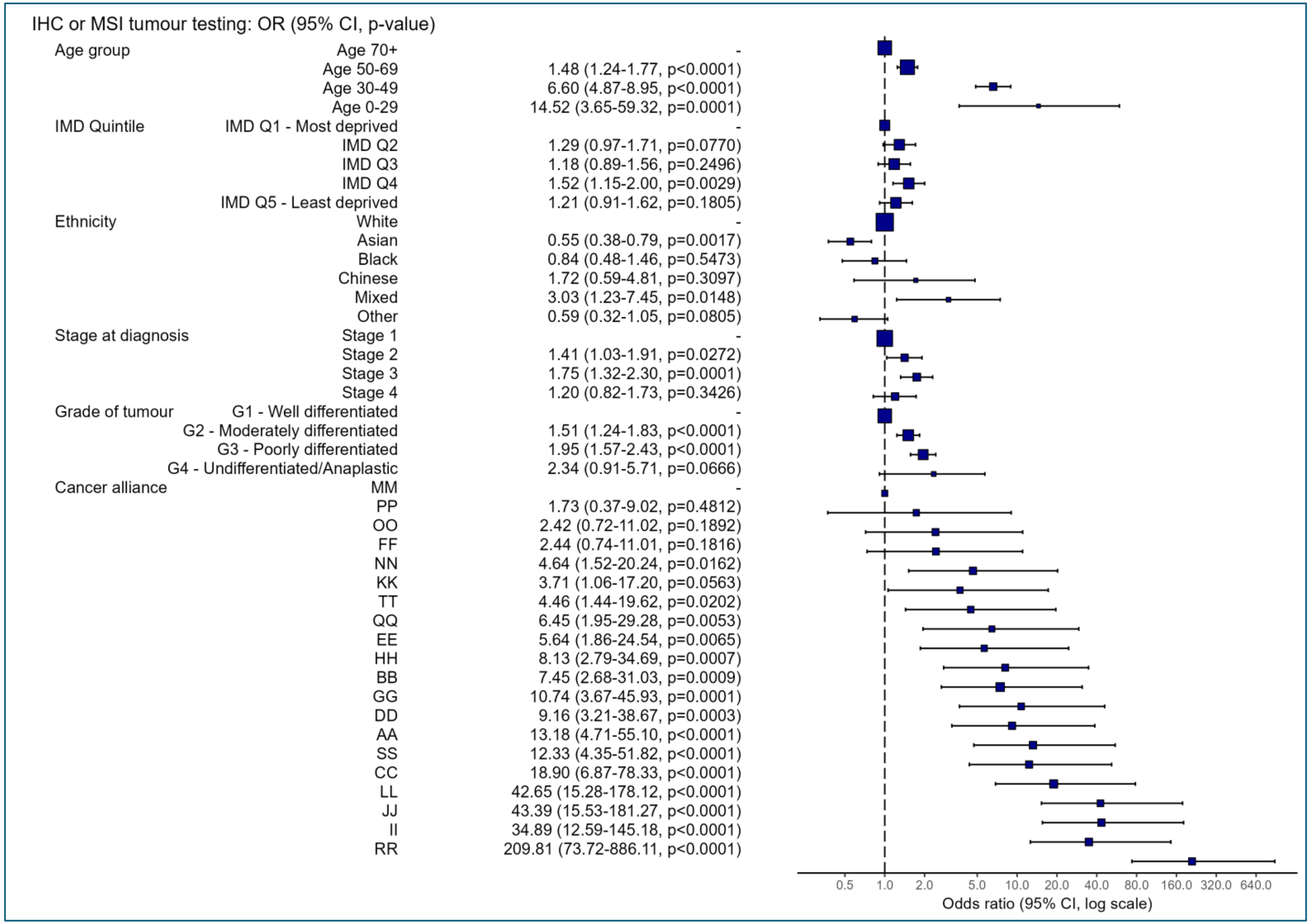

Supplement: online supplemental file 1 [file jmg-61-12-s001.pdf]
